# A novel glycolipid composite index predicting cardiovascular disease in Chinese adults with abnormal glucose metabolism: a nationwide cohort study

**DOI:** 10.3389/fcvm.2026.1791890

**Published:** 2026-05-14

**Authors:** Dan-dan Peng, Ping Zhu, Ji-chen Xie, Wei Yong, Kai Ye, Jun-jie Gao, Zhao-xia Shuai

**Affiliations:** Department of Clinical Laboratory, the Second Affiliated Hospital of Wannan Medical University, Wuhu, China

**Keywords:** abnormal glucose metabolism, cardiovascular disease, CHARLS, insulin resistance, TyG-GLM6

## Abstract

**Background:**

Cardiovascular disease (CVD) is the leading cause of mortality among individuals with abnormal glucose metabolism. Existing insulin resistance (IR) surrogate indexes show limited predictive capacity in Chinese populations and fail to capture comprehensive glycolipid metabolic dysregulation. We developed and validated TyG-GLM6, a composite index integrating six metabolic parameters (fasting glucose, triglycerides, HDL-C, LDL-C, Age, and BMI), and compared its predictive performance against nine conventional IR indexes.

**Methods:**

This prospective cohort study analyzed 3,684 participants aged ≥45 years with abnormal glucose metabolism from the China Health and Retirement Longitudinal Study (2011–2020). Associations between TyG-GLM6 and incident CVD were evaluated using multivariate logistic regression and restricted cubic splines. Seven machine learning algorithms were implemented, with performance assessed via ROC curves and SHAP analysis. External validation was conducted in 2,105 participants from a tertiary hospital.

**Results:**

During 9-year follow-up, 824 (22.4%) participants developed CVD. After full adjustment including biochemical markers, TyG-GLM6 was the only index retaining independent predictive significance (OR: 1.04, 95% CI: 1.01–1.08, *P* = 0.028), while eGDR, TyG-WC, and CVAI were attenuated to non-significance. TyG-GLM6 exhibited a linear dose-response relationship with CVD risk (*P* for nonlinea*r* = 0.768, *P* for overall < 0.001) and consistent performance across sex and age subgroups. Logistic regression achieved optimal performance (AUC: 0.587), with TyG-GLM6 among top predictors. External validation confirmed independent prediction (adjusted OR: 2.02, 95% CI: 1.84–2.20, *P* < 0.001).

**Conclusions:**

TyG-GLM6 demonstrates superior independent predictive value for CVD in Chinese adults with abnormal glucose metabolism, outperforming conventional IR indexes in fully adjusted models. Its linear dose-response relationship, demographic robustness, and external validation support its utility for early risk stratification and personalized prevention strategies. Validation in ethnically diverse populations is warranted.

## Introduction

Cardiovascular disease (CVD) remains the leading cause of morbidity and mortality worldwide, particularly among elderly populations, where it is characterized by high incidence, disability, and mortality rates, posing persistent challenges to public health systems and socioeconomic development (1, 2). In China, accelerated population aging coupled with lifestyle transitions has driven a rising prevalence of glucose metabolism disorders—including prediabetes and type 2 diabetes—among middle-aged and elderly individuals. Substantial evidence indicates that these populations face significantly elevated CVD risk (3–6). The underlying mechanisms are closely linked to multiple metabolic dysregulations, including insulin resistance (IR), dyslipidemia, and chronic low-grade inflammation, underscoring abnormal glucose metabolism as a critical CVD risk factor (7). Consequently, developing risk assessment tools that combine strong predictive performance with clinical practicality is essential for enabling early identification and targeted intervention in this high-risk population.

Currently, several IR surrogate indexes based on routine clinical parameters—such as the Triglyceride-Glucose Index (TyG) and estimated Glucose Disposal Rate (eGDR)—are utilized for CVD risk prediction (8–10). These indexes leverage readily available data from physical examinations, including lipid profiles and glucose levels, offering non-invasive, simple, and cost-effective advantages. Multiple studies have demonstrated their associations with CVD event risk (11, 12). However, existing indexes face notable limitations when applied to middle-aged and elderly Chinese populations with abnormal glucose metabolism. First, their predictive efficacy in this specific cohort remains inadequately validated, raising concerns regarding potential variations related to ethnicity and metabolic background. Second, most indexes focus on isolated metabolic pathways, failing to comprehensively capture the complex, synergistic dysregulation of glucose and lipid metabolism characteristic of this population, thereby limiting their predictive accuracy and clinical utility.

To address these research gaps, we developed a novel glycolipid composite index, TyG-GLM6 (Triglyceride-Glucose–Glycolipid Metabolic 6-index). Building upon the traditional TyG index, TyG-GLM6 integrates six key metabolic parameters, including fasting glucose, triglycerides, high-density lipoprotein cholesterol (HDL-C), low-density lipoprotein cholesterol (LDL-C), and body mass index, to provide a more comprehensive assessment of IR severity and overall glycolipid metabolic dysfunction (13). Using data from a large-scale nationwide prospective cohort, this study systematically evaluated the predictive capacity of TyG-GLM6 for CVD risk in Chinese middle-aged and elderly individuals with abnormal glucose metabolism and compared its discriminative performance against nine conventional IR surrogate indexes. Furthermore, by integrating machine learning approaches, we explored the predictive potential of TyG-GLM6 in complex multidimensional data contexts. This study aims to provide robust scientific evidence for early risk stratification, personalized interventions, and optimized cardiovascular health management strategies in this high-risk population.

## Methods

### Study design and population

The China Health and Retirement Longitudinal Study (CHARLS) is an ongoing, nationally representative longitudinal survey designed to assess the social, economic, and health status of Chinese adults aged 45 years and older. The CHARLS cohort was established using a multistage probability sampling strategy, with participants selected from 150 counties (districts) and 450 villages (communities) across 28 provinces nationwide (14). The baseline CHARLS survey was conducted in 2011, with subsequent follow-up waves in 2013, 2015, 2018, and 2020. The CHARLS protocol was approved by the Biomedical Ethics Review Committee of Peking University (IRB00001052-11015), and all participants provided written informed consent prior to enrollment. The study adheres to the principles of the Declaration of Helsinki. Detailed information about CHARLS is publicly available at http://charls.pku.edu.cn/en.

**Figure 1:**
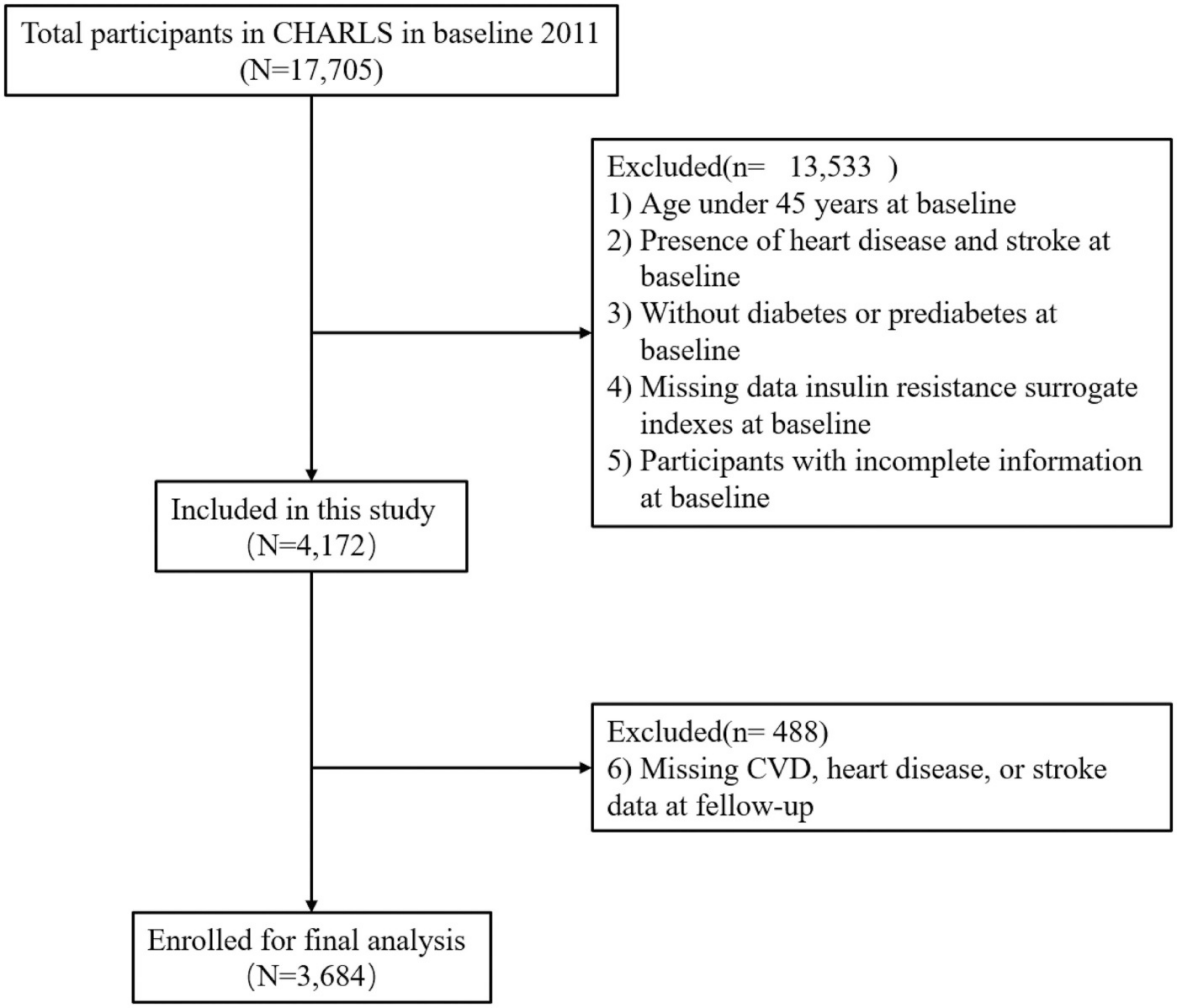
Flowchart of participant selection.

This prospective cohort study utilized data from all five CHARLS survey waves (2011–2020), with 2011 serving as baseline. Follow-up interviews were conducted biennially through standardized face-to-face visits by trained interviewers using computer-assisted technology to ensure data quality and consistency. The baseline survey enrolled 17,705 participants. Participants were sequentially excluded based on the following criteria: (1) age <45 years at baseline; (2) prevalent CVD (heart disease or stroke) or cancer at baseline; (3) absence of abnormal glucose metabolism (prediabetes or type 2 diabetes) at baseline; (4) missing data for TyG-GLM6 or any other IR surrogate index at baseline; (5) incomplete anthropometric, lifestyle, sociodemographic, or biochemical data at baseline; and (6) missing CVD outcome data during follow-up. After applying these criteria, 3,684 participants were included in the final analysis. The participant selection process is illustrated in Figure 1.

### Data collection and measurement

At baseline, trained interviewers collected data using standardized questionnaires, including: (1) demographic characteristics (sex, age, educational attainment, and marital status); (2) anthropometric measurements [systolic blood pressure (SBP), diastolic blood pressure (DBP), height, weight, and waist circumference (WC)]; (3) lifestyle factors (smoking and alcohol consumption status); (4) medical history (hypertension, diabetes, and heart disease); and (5) biochemical parameters [triglycerides (TG), total cholesterol (TC), high-density lipoprotein cholesterol (HDL-C), low-density lipoprotein cholesterol (LDL-C), serum creatinine (Scr), fasting plasma glucose (FPG), glycated hemoglobin A1c (HbA1c), serum uric acid (SUA), blood urea nitrogen (BUN), and C-reactive protein (CRP)].

Educational attainment was dichotomized as below high school vs. high school or above. Smoking status was categorized as current smoker or non-smoker. Alcohol consumption was classified as current drinker or non-drinker. Blood pressure was measured three times after participants rested in a seated position for 5 min, and the average of the three measurements was used for analysis. Venous blood samples were collected after an overnight fast of at least 8 h for all laboratory assessments.

### Definition of abnormal glucose metabolism

Prediabetes was defined as FPG 5.6–6.9 mmol/L (100–125 mg/dL) or HbA1c 5.7%–6.4% in the absence of a diabetes diagnosis. Type 2 diabetes was defined as self-reported physician diagnosis, use of glucose-lowering medication, FPG ≥7.0 mmol/L (126 mg/dL), or HbA1c ≥ 6.5%. Abnormal glucose metabolism was defined as the presence of either prediabetes or type 2 diabetes (15). Hypertension was defined as self-reported physician diagnosis, current use of antihypertensive medication, or measured SBP ≥140 mmHg or DBP ≥90 mmHg (16).

### Definition of TyG-GLM6 and other IR surrogate indexes

IR was assessed using several validated surrogate indexes derived from routine clinical parameters. The primary index evaluated was TyG-GLM6, which integrates six key metabolic parameters: fasting glucose, triglycerides, LDL-C, HDL-C, waist circumference, and body mass index (BMI). For comparative analysis, nine additional IR surrogate or glycolipid metabolism indexes were included: GLM6, eGDR, TyG, TyG-WC, TyG-BMI, TyG-WHtR, TG/HDL-C, METS-IR, and AIP. All indexes were calculated using formulas from previously published studies, with detailed computational methods provided in Supplementary Table S1.

### Outcome ascertainment

The primary outcome was incident CVD, defined as new-onset heart disease or stroke during follow-up. Participants self-reported physician-confirmed diagnoses of CVD at each follow-up wave (2013–2020), consistent with established CHARLS methodology. Incident CVD events were defined as new cases occurring between baseline (2011) and the most recent follow-up (2020) among participants free of CVD at baseline. CVD status was assessed using standardized questions at each wave: “Have you been diagnosed by a physician with heart disease/stroke?” or “Are you currently receiving treatment (traditional Chinese medicine, Western medicine, physical therapy, acupuncture, or occupational therapy) for heart disease/stroke?” The CHARLS research team implemented rigorous quality control procedures to ensure data accuracy and reliability (14).

### Missing data handling

Participants with missing data for IR surrogate indexes, CVD outcomes during follow-up, or baseline covariates (sociodemographic and health-related variables) were excluded from the analysis. To assess potential selection bias, baseline characteristics were compared between excluded and included participants.

### Machine learning model development and SHAP interpretability analysis

Seven machine learning algorithms were evaluated: Logistic Regression (LR), Decision Tree (DT), Random Forest (RF), Support Vector Machine (SVM), XGBoost, LightGBM, and Deep Neural Network (DNN). LR estimates binary event probabilities through linear combinations and a sigmoid function; DT partitions data through recursive splitting rules, offering high interpretability but susceptibility to overfitting; RF aggregates multiple decision trees to reduce variance and enhance robustness against noise and outliers; SVM performs classification using maximum margin principles and kernel functions; XGBoost and LightGBM are gradient boosting frameworks that iteratively optimize weak learners—XGBoost excels in handling bias-variance tradeoffs, while LightGBM improves computational efficiency through histogram-based optimization; DNN automatically extracts features through multilayer nonlinear transformations.

All continuous features were standardized to eliminate scale effects. Model hyperparameters were optimized using five-fold cross-validation combined with grid search and manual fine-tuning, with area under the curve (AUC) as the optimization metric. To elucidate model decision-making mechanisms, we employed the SHapley Additive exPlanations (SHAP) framework based on cooperative game theory. SHAP quantifies the marginal contribution of each feature to model predictions by assigning SHAP values, thereby enhancing model interpretability.

### Model performance evaluation

Model performance was assessed using receiver operating characteristic (ROC) curves, AUC, sensitivity, specificity, accuracy, and F1 score. Pairwise comparisons of AUC values were conducted using the DeLong test. Decision curve analysis (DCA) and calibration curves were employed to evaluate clinical utility and calibration of the final model. Model calibration was assessed using the Hosmer–Lemeshow test, with *P* > 0.05 indicating acceptable agreement between predicted and observed event rates.

### Statistical analysis

All statistical analyses were performed using R software (version 4.4.3), and machine learning models were developed using the Python scikit-learn library. Two-tailed *P* < 0.05 was considered statistically significant. Continuous variables are presented as mean ± standard deviation (SD) or median [interquartile range (IQR)] according to their distribution, while categorical variables are presented as frequency (percentage). Between-group comparisons for normally distributed continuous variables were performed using independent *t*-tests or one-way analysis of variance (ANOVA); non-normally distributed continuous variables were compared using Mann–Whitney *U* tests. Categorical variables were compared using chi-square tests. Logistic regression models were used to assess associations between TyG-GLM6 and other glycolipid metabolism indexes with incident CVD risk. Cox proportional hazards regression models were conducted as complementary survival analyses. Time-to-event was calculated from baseline (2011) to the first occurrence of CVD or the last follow-up. Schoenfeld residuals and graphical tests were used to examine the proportional hazards assumption. Hazard ratios (HRs) and 95% confidence intervals (CIs) were estimated for each metabolic index.

## Results

### Baseline characteristics of study participants

This study included 3,684 participants with abnormal glucose metabolism. During the median follow-up of 9 years (2011–2020), 824 (22.4%) participants developed CVD, while 2,860 (77.6%) remained free of CVD. Baseline characteristics stratified by incident CVD status are presented in Table 1. Compared with participants who did not develop CVD, those with incident CVD were significantly older and had higher values for weight, BMI, WC, BUN, fasting glucose, TG, HbA1c, and all IR surrogate indexes (GLM6, TyG, TyG-GLM6, TyG-WC, TyG-BMI, TyG-WHtR, TG/HDL-C, METS-IR, AIP, CVAI, NHHR, and UHR), as well as lower eGDR and HDL-C levels (all *P* < 0.05). The proportion of males, current alcohol drinkers, and individuals with hypertension was also significantly higher in the CVD group (all *P* < 0.05). No significant differences were observed between groups in height, serum creatinine, total cholesterol, LDL-C, CRP, uric acid, educational attainment, or smoking status (all *P* > 0.05).

**Table 1:** Baseline characteristics of the study participants.

Variables	Total (*n* = 3,684)	Non-CCVD (*n* = 2,860)	New onset CCVD (*n* = 824)	*P*
Age, (years)	59.60 ± 9.14	59.39 ± 9.24	60.33 ± 8.75	**0**.**010**
Gender, n(%)				**0**.**004**
Female	1,935 (52.52)	1,466 (51.26)	469 (56.92)	
Male	1,749 (47.48)	1,394 (48.74)	355 (43.08)	
Education, n(%)				0.600
High school and below	3,637 (98.72)	2,825 (98.78)	812 (98.54)	
High school above	47 (1.28)	35 (1.22)	12 (1.46)	
Smoking, n(%)				0.058
No	2,256 (61.24)	1,728 (60.42)	528 (64.08)	
Yes	1,428 (38.76)	1,132 (39.58)	296 (35.92)	
Drinking, n(%)				**0**.**012**
No	2,404 (65.26)	1,836 (64.20)	568 (68.93)	
Yes	1,280 (34.74)	1,024 (35.80)	256 (31.07)	
Hypertension, n(%)				**<**.**001**
No	2,531 (68.70)	2,006 (70.14)	525 (63.71)	
Yes	1,153 (31.30)	854 (29.86)	299 (36.29)	
Height, (cm)	157.89 ± 8.74	157.94 ± 8.61	157.72 ± 9.21	0.517
Weight, (cm)	59.55 ± 11.59	59.00 ± 11.18	61.44 ± 12.72	**<**.**001**
BMI	23.82 ± 3.96	23.59 ± 3.77	24.63 ± 4.46	**<**.**001**
WC, (cm)	85.45 ± 11.91	84.85 ± 11.58	87.54 ± 12.78	**<**.**001**
BUN, (mg/dL)	15.95 ± 4.64	16.04 ± 4.69	15.67 ± 4.45	**0**.**043**
GLU, (mg/dL)	121.20 ± 38.65	120.30 ± 37.55	124.35 ± 42.12	**0**.**013**
CREA, (mg/dL)	0.79 ± 0.27	0.79 ± 0.28	0.77 ± 0.18	0.077
CHO, (mg/dL)	198.93 ± 39.67	198.38 ± 39.63	200.86 ± 39.77	0.113
TG, (mg/dL)	142.64 ± 108.28	140.36 ± 109.78	150.57 ± 102.55	**0**.**017**
HDL-C, (mg/dL)	50.78 ± 15.62	51.26 ± 15.61	49.12 ± 15.55	**<**.**001**
LDL-C, (mg/dL)	119.48 ± 37.24	118.97 ± 36.92	121.24 ± 38.30	0.123
CRP, (mg/L)	2.85 ± 7.72	2.80 ± 7.87	3.00 ± 7.19	0.531
HBA1c, (%)	5.46 ± 0.98	5.43 ± 0.96	5.55 ± 1.04	**0**.**003**
UA, (mg/dL)	4.50 ± 1.26	4.51 ± 1.27	4.47 ± 1.25	0.421
GLM6	5.70 ± 0.40	5.68 ± 0.40	5.78 ± 0.41	**<**.**001**
eGDR	9.39 ± 2.16	9.51 ± 2.11	8.99 ± 2.26	**<**.**001**
TyG	8.85 ± 0.66	8.82 ± 0.65	8.93 ± 0.69	**<**.**001**
TyG-GLM6	50.69 ± 7.17	50.34 ± 7.04	51.91 ± 7.47	**<**.**001**
TyG-WC	757.70 ± 131.67	750.28 ± 128.48	783.46 ± 139.22	**<**.**001**
TyG-BMI	211.44 ± 42.13	208.77 ± 40.46	220.71 ± 46.31	**<**.**001**
TyG-WHtR	4.81 ± 0.85	4.76 ± 0.83	4.98 ± 0.89	**<**.**001**
TG/HDL-C	3.56 ± 4.75	3.47 ± 4.72	3.89 ± 4.85	**0**.**025**
METS-IR	6.90 ± 0.54	6.88 ± 0.54	6.99 ± 0.55	**<**.**001**
AIP	0.89 ± 0.80	0.86 ± 0.80	1.00 ± 0.80	**<**.**001**
CVAI	99.29 ± 44.12	96.35 ± 43.35	109.52 ± 45.26	**<**.**001**
NHHR	3.26 ± 1.56	3.20 ± 1.52	3.46 ± 1.65	**<**.**001**
UHR	0.10 ± 0.05	0.10 ± 0.05	0.10 ± 0.06	**0**.**015**

Bold value indicates *P* < 0.05.

### Association of IR surrogate indexes with incident CVD

To examine associations between IR surrogate indexes and incident CVD among participants with abnormal glucose metabolism, univariate and multivariate logistic regression analyses were performed (Table 2). Univariate analysis revealed that GLM6 (OR: 1.88, 95% CI: 1.68–2.11), eGDR (OR: 0.89, 95% CI: 0.86–0.92), TyG (OR: 1.28, 95% CI: 1.17–1.40), TyG-GLM6 (OR: 1.03, 95% CI: 1.02–1.04), TyG-WC (OR: 1.01, 95% CI: 1.01–1.01), TyG-BMI (OR: 1.01, 95% CI: 1.00–1.01), TyG-WHtR (OR: 1.36, 95% CI: 1.23–1.51), TG/HDL-C (OR: 1.02, 95% CI: 1.01–1.03), AIP (OR: 1.23, 95% CI: 1.13–1.34), CVAI (OR: 1.01, 95% CI: 1.01–1.01), NHHR (OR: 1.11, 95% CI: 1.08–1.15), and UHR (OR: 6.17, 95% CI: 4.76–7.99) were all significantly associated with incident CVD (all *P* < 0.05). In multivariate analysis adjusting for demographic characteristics, lifestyle factors, and anthropometric measurements, eGDR (OR: 0.94, 95% CI: 0.90–0.99), TyG-GLM6 (OR: 1.02, 95% CI: 1.00–1.03), TyG-WC (OR: 0.99, 95% CI: 0.99–1.00), and CVAI (OR: 1.01, 95% CI: 1.00–1.01) remained independently associated with incident CVD (all *P* < 0.05). These findings indicate that the novel glycolipid composite index TyG-GLM6 is significantly associated with CVD risk in this population.

**Table 2:** Univariate and multivariate regression analysis of potential indicators.

Variables	Univariate					Multivariate				
	*β*	S.E	*Z*	*P*	OR (95%CI)	*β*	S.E	*Z*	*P*	OR (95%CI)
GLM6	0.63	0.10	6.36	**<**.**001**	1.88 (1.55–2.29)					
eGDR	−0.11	0.02	−6.12	**<**.**001**	0.89 (0.86–0.93)	−0.06	0.02	−2.54	**0**.**011**	0.94 (0.90–0.99)
TyG	0.25	0.06	4.29	**<**.**001**	1.28 (1.14–1.44)					
TyG-GLM6	0.03	0.01	5.52	**<**.**001**	1.03 (1.02–1.04)	0.02	0.01	2.60	**0**.**009**	1.02 (1.01–1.03)
TyG-WC	0.01	0.00	6.35	**<**.**001**	1.01 (1.01–1.01)	−0.01	0.00	−2.41	**0**.**016**	0.99 (0.99–0.99)
TyG-BMI	0.01	0.00	7.00	**<**.**001**	1.01 (1.01–1.01)					
TyG-WHtR	0.31	0.05	6.42	**<**.**001**	1.36 (1.24–1.50)					
TG/HDL	0.02	0.01	2.21	**0**.**027**	1.02 (1.01–1.03)					
AIP	0.20	0.05	4.18	**<**.**001**	1.23 (1.11–1.35)					
CVAI	0.01	0.00	7.53	**<**.**001**	1.01 (1.01–1.01)	0.01	0.00	4.28	**<**.**001**	1.01 (1.01–1.01)
NHHR	0.10	0.02	4.15	**<**.**001**	1.11 (1.05–1.16)					
UHR	1.82	0.76	2.39	**0**.**017**	6.17 (1.39–27.51)					

OR, odds ratio; CI, confidence interval.

Bold value indicates *P* < 0.05.

### Dose–response relationships between IR surrogate indexes and incident CVD

Table 3 presents the associations between the four significant multivariate predictors (TyG-GLM6, eGDR, TyG-WC, and CVAI) and incident CVD across three progressively adjusted models. In the unadjusted model (Model 1), all four indexes were significantly associated with CVD risk (all *P* < 0.001). After adjustment for demographic characteristics, lifestyle factors, and anthropometric measurements (Model 2), TyG-GLM6 (OR: 1.02, 95% CI: 1.01–1.03, *P* = 0.007), eGDR (OR: 0.94, 95% CI: 0.90–0.99, *P* = 0.017), TyG-WC (OR: 1.01, 95% CI: 1.01–1.01, *P* = 0.024), and CVAI (OR: 1.01, 95% CI: 1.01–1.01, *P* = 0.027) all retained statistical significance. However, after further adjustment for all biochemical markers (Model 3), only TyG-GLM6 remained independently associated with incident CVD (OR: 1.04, 95% CI: 1.01–1.08, *P* = 0.028), while the other three indexes were attenuated to non-significance.

**Table 3:** Multivariate regression analysis of the associations between potential indicators and cardiovascular diseases in individuals with abnormal glucose metabolism.

Variables	Model1		Model2		Model3	
	OR (95%CI)	*P*	OR (95%CI)	*P*	OR (95%CI)	*P*
TyG-GLM6	1.03 (1.02–1.04)	**<**.**001**	1.02 (1.01–1.03)	**0**.**007**	1.04 (1.01–1.08)	**0**.**028**
eGDR	0.89 (0.86–0.93)	**<**.**001**	0.94 (0.90–0.99)	**0**.**017**	0.93 (0.76–1.14)	0.493
TyG-WC	1.01 (1.01–1.01)	**<**.**001**	1.01 (1.01–1.01)	**0**.**024**	1.00 (1.00–1.01)	0.217
CVAI	1.01 (1.01–1.01)	**<**.**001**	1.01 (1.01–1.01)	**0**.**027**	1.00 (1.00–1.01)	0.103

Model 1: was unsdjusted.

Model 2: was adjusted for age, gender, education, smoking, drinking, height, weight, BMI, Hypertension, WC.

Model 3: was adjusted for Model2 + BUN, GLU, CREA, CHO, TG, HDL-C, LDL-C, CRP, HBA1c, UA.

OR, odds ratio; CI, confidence interval; CCVD, Cardiovascular and Cerebrovascular Diseases; BMI, body mass index; WBC, white blood cell; PLT, platelet; BUN, blood urea nitrogen; FBG, fasting blood glucose; Cr, creatinine; TC, total cholesterol; TG, triglycerides; HDL-C, high-density lipoprotein cholesterol; LDL-C, low-density lipoprotein cholesterol; CRP, C-reactive protein; HbA1C, glycated hemoglobin; UA, uric acid; GLM6, glycolipid metabolism 6 factors; TyG, triglyceride-glucose index; CVAI, Chinese visceral adiposity index.

Bold value indicates *P* < 0.05.

**Figure 2:**
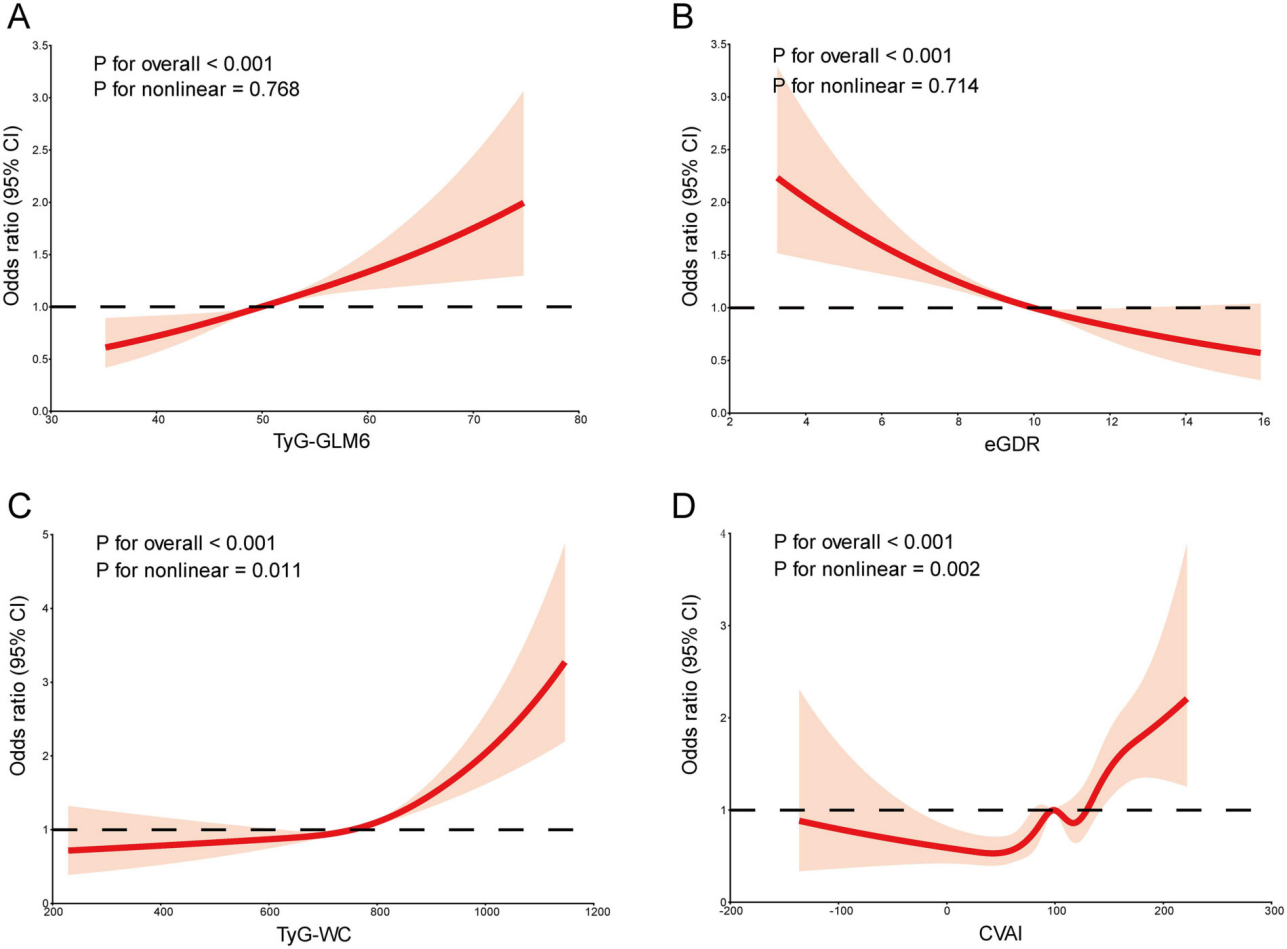
**(A–D)** Dose–response relationships between IR surrogate indexes and incident CVD risk using restricted cubic spline analysis.

Restricted cubic spline (RCS) analyses were conducted to examine dose–response relationships between the four indexes and incident CVD (Figure 2). After adjusting for potential confounders, TyG-GLM6 exhibited a linear positive association with CVD risk (*P* for nonlinear = 0.768, *P* for overall <0.001), while eGDR showed a linear negative association (*P* for nonlinear = 0.714, *P* for overall <0.001). In contrast, TyG-WC (*P* for nonlinear = 0.011, *P* for overall <0.001) and CVAI (*P* for nonlinear = 0.002, *P* for overall <0.001) demonstrated significant nonlinear associations with CVD risk. Overall, RCS findings were consistent with the multivariate regression results.

### Subgroup analyses of IR surrogate indexes and incident CVD

**Figure 3:**
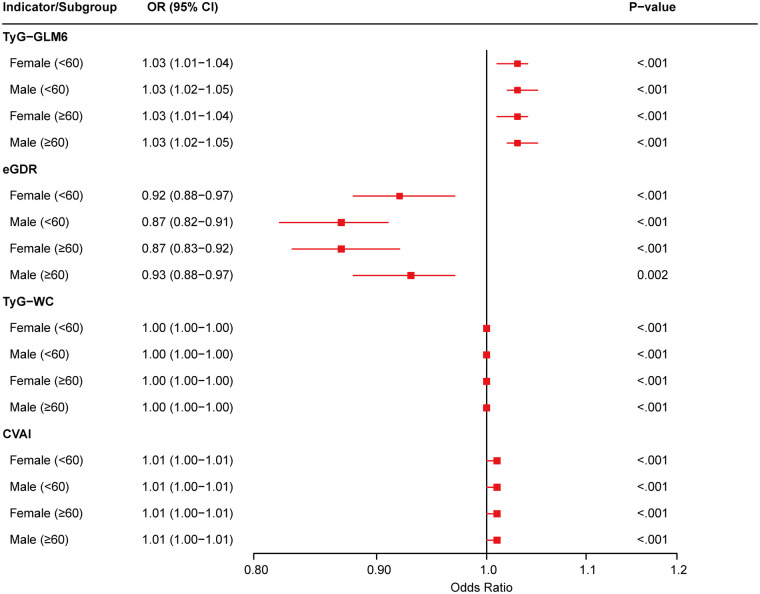
Subgroup analyses of associations between IR surrogate indexes and incident CVD stratified by sex and age.

To further explore the robustness of associations between the four IR surrogate indexes and incident CVD, subgroup analyses stratified by sex and age were performed (Figure 3). The direction and magnitude of associations between all four indexes and CVD risk remained consistent across subgroups. No significant interactions were observed between the indexes and sex or age (all *P* interaction >0.05), suggesting that the predictive value of these glycolipid metabolism indexes for CVD events is stable across different demographic strata.

### Survival analysis using Cox proportional hazards regression

Univariate Cox regression showed that all metabolic indicators were significantly associated with incident CVD (all *P* < 0.001). Specifically, TyG-GLM6 (HR = 1.026, 95% CI: 1.017–1.036, *P* < 0.001), eGDR (HR = 0.905, 95% CI: 0.878–0.934, *P* < 0.001), TyG-WC (HR = 1.002, 95% CI: 1.001–1.002, *P* < 0.001), and CVAI (HR = 1.007, 95% CI: 1.005–1.008, *P* < 0.001) were all strongly associated with CVD risk. In the multivariable-adjusted model including all four indices, TyG-GLM6 (HR = 1.017, 95% CI: 1.004–1.030, *P* = 0.011), eGDR (HR = 0.948, 95% CI: 0.911–0.986, *P* = 0.009), TyG-WC (HR = 0.998, 95% CI: 0.997–1.000, *P* = 0.010), and CVAI (HR = 1.007, 95% CI: 1.004–1.011, *P* < 0.001) remained independently associated with incident CVD. Kaplan–Meier survival curves stratified by TyG-GLM6 median split showed significantly worse CVD-free survival in the high TyG-GLM6 group (log-rank *P* < 0.001). The proportional hazards assumption was generally satisfied. These survival analyses confirmed the robustness of our primary findings (Supplementary Table S2).

### Machine learning-based CVD risk prediction

**Figure 4:**
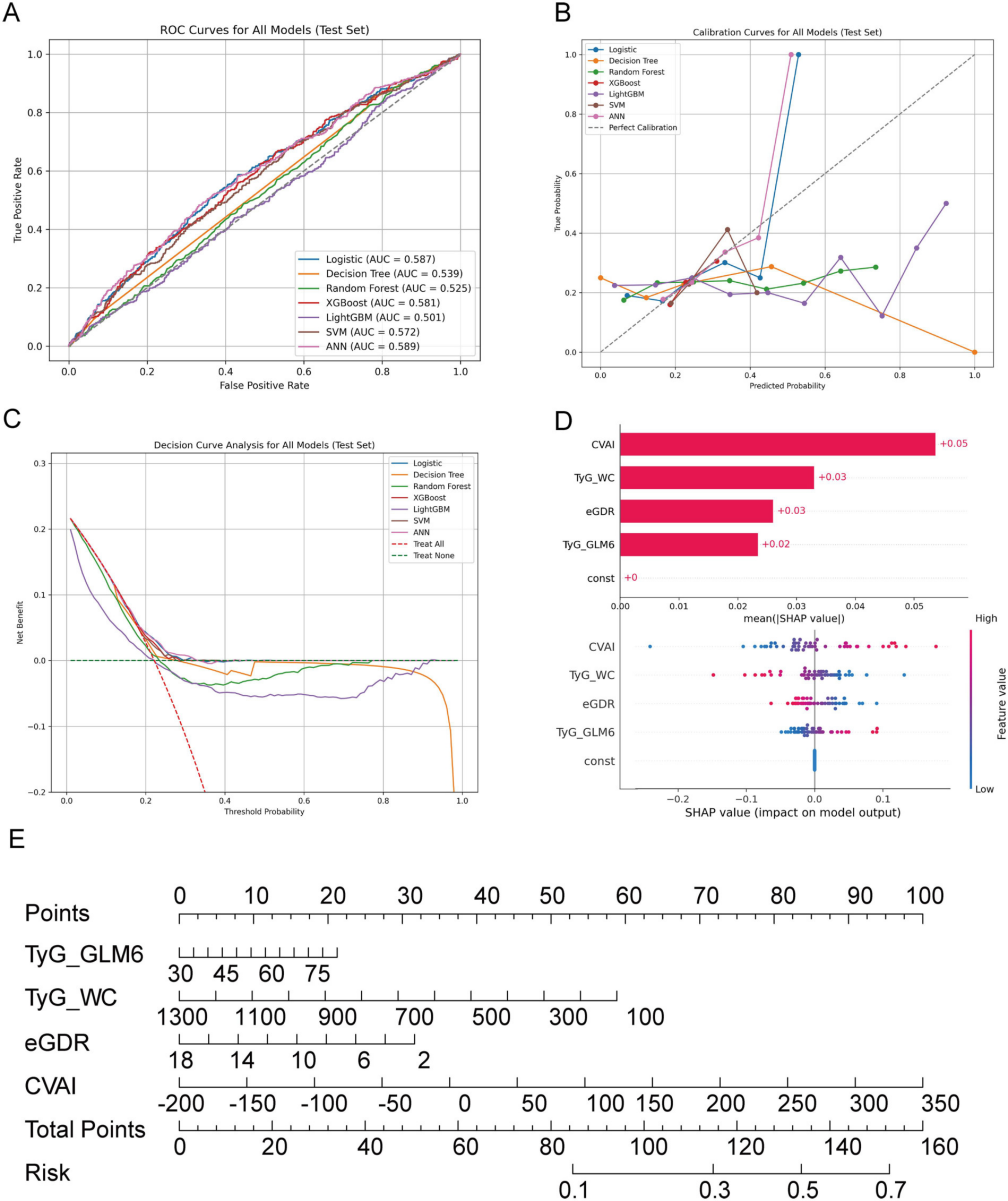
Machine learning-based CVD risk prediction model performance and feature importance analysis. **(A)** Receiver operating characteristic (ROC) curves for seven machine learning models on the test set. The area under the curve (AUC) value for each model is annotated next to its respective curve. The dashed grey line represents a classifier with no discriminative power (AUC = 0.5). **(B)** Calibration curves for the models on the test set. The diagonal dashed line represents perfect calibration, where predicted probabilities perfectly match observed event frequencies. The degree of deviation from this line for each model's curve reflects its calibration error. **(C)** Decision curve analysis (DCA) for the models on the test set. The *y*-axis represents the net benefit calculated across a range of threshold probabilities (*x*-axis). The grey solid line assumes no patients have the event (“Treat None”), and the solid black line assumes all patients have the event (“Treat All”). A model curve higher than these lines indicates clinical utility across that threshold range. **(D)** Bar chart displays the average absolute SHAP value of each feature, and its height directly reflects the global importance of the feature to the prediction result. Bee-swarm plot further reveals details through sample level distribution, where each point represents the SHAP value of a single sample, with red indicating high eigenvalues and blue indicating low eigenvalues. **(E)** Logistic regression nomogram, used to calculate total points based on individual feature values and map them to risk probabilities, supporting rapid assessment of disease risk in clinical practice.

Seven machine learning algorithms were employed to develop CVD risk prediction models. Performance evaluation in the validation set (Figures 4A–C) revealed that logistic regression (LR) achieved an AUC of 0.587, comparable to or exceeding that of more complex algorithms including Random Forest (AUC: 0.582), XGBoost (AUC: 0.579), and Deep Neural Network (AUC: 0.574).Considering the comparable discriminative performance, LR was selected as the optimal algorithm due to its superior interpretability, computational efficiency, and clinical applicability. Unlike “black-box” models, LR provides transparent coefficient estimates that facilitate clinical understanding and trust. Furthermore, LR's simplicity enhances implementability in diverse clinical settings, including resource-limited primary care environments. Feature importance analysis identified CVAI, TyG-WC, eGDR, and TyG-GLM6 as the most influential predictors (Figure 4D). Based on these results, we constructed a nomogram visualization of the LR model coefficients (Figure 4E) to facilitate individualized risk quantification in clinical practice. Furthermore, we developed an interactive web-based prediction tool (cardiovascular-risk-tool, available at https://gjj1995.github.io/cardiovascular-risk-tool/) that allows clinicians to input patients' glycolipid metabolism parameters and obtain real-time CVD risk estimates. The source code and interactive interface files are provided in Supplementary Table S3.

### External validation of TyG-GLM6 in an independent clinical cohort

To evaluate the clinical utility of TyG-GLM6, we conducted external validation in an independent cohort comprising 2,105 participants recruited from the Second Affiliated Hospital of Wannan medical college between 2020 and 2024. The validation cohort included 1,059 hospitalized patients with abnormal glucose metabolism and diagnosed CVD, and 1,046 individuals with abnormal glucose metabolism but free of CVD identified from routine health examination records. Baseline characteristics revealed significantly higher TyG-GLM6 values in the CVD group compared with the non-CVD group (*P* < 0.001) (Supplementary Table S4 and Table 4). Both univariate and multivariate logistic regression analyses confirmed that TyG-GLM6 was a significant independent predictor of CVD in this clinical cohort (adjusted OR: 2.02, 95% CI: 1.84–2.20, *P* < 0.001), corroborating the findings from the CHARLS cohort.

**Table 4:** Univariate and multivariate logistic regression analysis of clinical data.

Variables	Univariate					Multivariate				
	*β*	S.E	*Z*	*P*	OR (95%CI)	*β*	S.E	*Z*	*P*	OR (95%CI)
HbA1c	0.36	0.03	10.68	**<**.**001**	1.44 (1.34–1.54)	0.39	0.04	9.81	**<**.**001**	1.47 (1.36–1.59)
GLU	0.10	0.02	5.77	**<**.**001**	1.10 (1.07–1.14)					
TG	0.02	0.05	0.40	0.689	1.02 (0.92–1.13)					
LDL	−0.69	0.06	−12.32	**<**.**001**	0.50 (0.45–0.56)	−1.59	0.09	−17.72	**<**.**001**	0.20 (0.17–0.24)
HDL	−2.31	0.17	−13.53	**<**.**001**	0.10 (0.07–0.14)					
TyG	0.16	0.08	2.17	**0**.**030**	1.18 (1.02–1.37)	−6.03	0.42	−14.37	**<**.**001**	0.00 (0.00–0.01)
TyG-GLM6	0.03	0.01	3.61	**<**.**001**	1.03 (1.01–1.04)	0.70	0.05	15.30	**<**.**001**	2.02 (1.84–2.20)

OR, odds ratio; CI, confidence interval.

Bold value indicates *P* < 0.05.

## Discussion

This prospective cohort study systematically evaluated the predictive capacity of a novel glycolipid composite index, TyG-GLM6, for incident CVD among 3,684 Chinese middle-aged and elderly individuals with abnormal glucose metabolism enrolled in the China Health and Retirement Longitudinal Study (CHARLS). Our findings revealed a significant positive association between TyG-GLM6 and incident CVD risk (adjusted OR: 1.02, 95% CI: 1.01–1.03, *P* = 0.007), with TyG-GLM6 retaining independent predictive significance even after comprehensive adjustment for biochemical markers. This discovery provides a novel assessment tool for early risk identification in this high-risk population and holds important implications for clinical translation.

The mechanisms underlying the markedly elevated CVD risk in individuals with abnormal glucose metabolism are complex and multifactorial, involving interrelated pathophysiological processes including IR, dyslipidemia, endothelial dysfunction, and chronic low-grade inflammation (17, 18). Previous studies indicate that traditional single metabolic markers, such as fasting glucose or individual lipid parameters, often fail to comprehensively capture this intricate metabolic dysregulation (19). In recent years, IR surrogate indexes exemplified by TyG have demonstrated promising utility in CVD risk prediction (20–22), although their applicability across diverse populations requires further validation. The TyG-GLM6 index integrates six key metabolic parameters—fasting glucose, triglycerides, HDL-C, LDL-C, and BMI—to provide a more comprehensive assessment of the synergistic dysregulation of glucose and lipid metabolism characteristic of this population.

Our findings align with and extend previous international research. Zhou et al. reported that in the Iranian population, each 1 SD increase in TyG index was associated with 1.19-fold higher coronary heart disease risk and 1.16-fold higher CVD risk (23). whereas TyG-GLM6 demonstrated more robust predictive performance in our study. Sánchez-Íñigo et al. found that TyG-BMI predicted CVD events in Spanish patients with metabolic syndrome, although its independent predictive capacity was attenuated after multivariate adjustment (24). In contrast, TyG-GLM6 retained statistical significance in our fully adjusted model (Model 3), suggesting potentially superior predictive stability. These discrepancies may reflect differences in genetic backgrounds, lifestyle factors, and metabolic heterogeneity across populations, underscoring the importance of population-specific validation of metabolic risk indexes.

RCS analysis further revealed a linear positive dose–response relationship between TyG-GLM6 and CVD risk, providing important evidence for clinical risk stratification. Unlike the nonlinear associations observed for TyG-WC and CVAI, the linearity of the TyG-GLM6 relationship enhances its interpretability and practical utility in clinical settings (25). Furthermore, the presence of a clear dose–response relationship strengthens the evidence for a potential temporal association between TyG-GLM6 and CVD incidence, although causality cannot be definitively established in observational designs.

Subgroup analyses demonstrated that TyG-GLM6 maintained consistent predictive performance across sex and age strata, with no significant interactions observed (*P* for interaction > 0.05), indicating robust applicability and stability across demographic subgroups. This finding has important clinical implications, suggesting that TyG-GLM6 can serve as a universal risk assessment tool without requiring complex subgroup-specific adjustments. Particularly in middle-aged and elderly populations with abnormal glucose metabolism, the stable performance of TyG-GLM6 across subgroups provides a scientific foundation for developing standardized prevention strategies.

Machine learning analyses further validated the predictive capacity of TyG-GLM6 in complex multidimensional data contexts. Among seven algorithms evaluated, logistic regression achieved optimal overall performance with an AUC of 0.587. While this AUC is modest, it retains clinical utility given the inherent complexity of CVD prediction and inevitable information limitations in large-scale cohort studies. SHAP interpretability analysis identified CVAI, TyG-WC, eGDR, and TyG-GLM6 as the most influential predictors, further supporting the critical role of glycolipid metabolism indexes in CVD risk assessment. Based on these machine learning results, we developed an interactive web-based risk calculator to facilitate clinical implementation. Machine learning analyses validated the predictive capacity of TyG-GLM6 in complex multidimensional contexts. Notably, logistic regression achieved optimal overall performance, demonstrating that simpler, interpretable algorithms can match or exceed more complex models in this clinical prediction task. This finding has important practical implications: it suggests that transparent, easily interpretable models should be prioritized in clinical implementation, as they enhance clinician understanding, facilitate patient communication, and support trust in algorithmic decision support. The comparable performance of logistic regression and complex algorithms also suggests that the relationship between metabolic parameters and CVD risk in this population may be adequately captured by linear and additive effects, without requiring complex non-linear interactions. This parsimony enhances clinical applicability and generalizability.

External validation in an independent clinical cohort of 2,105 participants further confirmed the clinical utility of TyG-GLM6. Comparing individuals with abnormal glucose metabolism and prevalent CVD to those without CVD, TyG-GLM6 demonstrated significant discrimination (*P* < 0.001). Multivariate analysis confirmed TyG-GLM6 as an independent predictor of CVD (adjusted OR: 2.02, 95% CI: 1.84–2.20, *P* < 0.001), corroborating the CHARLS cohort findings. This external validation substantially enhances the reliability and generalizability of our results.

Arterial stiffness and pulse wave velocity as complementary vascular risk biomarkers Beyond metabolic parameters, mechanical vascular impairment—particularly arterial stiffness quantified by pulse wave velocity (PWV)—has emerged as a powerful independent predictor of CVD events (26, 27). PWV directly measures the structural and functional integrity of the arterial wall, reflecting cumulative vascular damage from chronic metabolic stress, oxidative injury, inflammation, and advanced glycation end-products. Recent evidence demonstrates synergistic relationships between glycolipid metabolism indexes and arterial stiffness. In individuals with prediabetes, TG/HDL-C ratio was independently associated with elevated PWV, outperforming TyG index in predicting vascular stiffness (26). Furthermore, higher PWV was associated with progressively increasing CVD risk in individuals stratified by SCORE2-Diabetes, confirming its independent prognostic value (27). These findings suggest that integrating metabolic indices (such as TyG-GLM6) with vascular functional biomarkers (such as PWV) may provide complementary risk information: metabolic indices capture systemic biochemical dysregulation, while PWV quantifies cumulative end-organ vascular damage. Combined assessment may enhance risk stratification accuracy, particularly in intermediate-risk populations. Future research should explore whether TyG-GLM6 correlates with PWV in Chinese populations with abnormal glucose metabolism, and whether combined models incorporating both metabolic and vascular stiffness parameters achieve superior discriminative performance (AUC ≥0.70). Such integrative approaches align with precision medicine principles and may enable more personalized CVD prevention strategies. However, practical implementation must consider the availability, cost, and standardization of PWV measurement in diverse clinical settings.

The superior predictive performance of TyG-GLM6 can be explained within a systems-medicine framework: chronic cardiometabolic diseases arise from integrated metabolic, vascular, immune, and inflammatory networks, with oxidative stress and chronic low-grade inflammation as central hubs. Unlike single markers that reflect isolated disturbances, TyG-GLM6 integrates six parameters covering glucose, lipid, and adiposity status, better capturing cumulative network-level dysregulation in individuals with abnormal glucose metabolism and aligning with the systemic pathogenesis of CVD, thus yielding stronger independent predictive value. This systems view is supported by recent research highlighting network dysregulation across metabolic, inflammatory, and vascular domains as a rationale for composite indices rather than single markers (28). Mechanistically, mitochondrial dysfunction acts as a key node linking oxidative stress, inflammation, aging, and cardiometabolic progression (29), chronic metabolic stress amplifies oxidative and inflammatory cascades via mitochondrial impairment, creating self-reinforcing cycles of metabolic disorder. The parameters in TyG-GLM6 represent downstream consequences of such mitochondrial and network dysfunction, supporting its ability to indirectly reflect cumulative mitochondrial and oxidative burden in high-risk populations.

This study has several notable strengths. First, it leveraged a large-scale, nationally representative prospective cohort with extended follow-up, providing a robust foundation for examining temporal associations. Second, by focusing on middle-aged and elderly individuals with abnormal glucose metabolism, the study targeted a clinically relevant high-risk population with clear implications for preventive interventions. Third, comprehensive analytical approaches integrating traditional statistical methods with machine learning algorithms yielded robust and internally consistent findings. Fourth, external validation in an independent clinical cohort enhanced the generalizability of the results. Finally, the development of an accessible online prediction tool facilitates translation of research findings into clinical practice.

While TyG-GLM6 demonstrated statistically significant and independent associations with CVD risk, the modest AUC values (0.587 in machine learning models) warrant explicit acknowledgment and contextual interpretation. Several factors contribute to this modest discriminative performance. First, CVD is a multifactorial endpoint influenced by complex interactions among genetic, environmental, behavioral, and clinical factors, many of which were unmeasured in our study. Second, reliance on self-reported CVD outcomes may introduce outcome misclassification. Third, metabolic indices—while capturing important pathophysiological processes—represent only one dimension of the multifaceted CVD risk landscape.

Importantly, TyG-GLM6 should be interpreted as a practical tool for risk stratification and enrichment within high-risk populations (individuals with abnormal glucose metabolism), rather than as a standalone screening or diagnostic marker for the general population. Its primary clinical utility lies in refining risk assessment beyond traditional risk factors, identifying individuals who may benefit from intensified preventive interventions, and serving as a simple, cost-effective adjunct to existing risk prediction frameworks. The linear dose-response relationship and demographic robustness further support its utility for population-level risk stratification in resource-limited settings.

Several important limitations warrant consideration. First, the observational design precludes definitive causal inference. While the prospective design, extended follow-up, dose-response relationship, and temporal sequence (exposure preceding outcome) support a potential causal association between TyG-GLM6 and CVD, residual confounding and reverse causation cannot be definitively excluded. Randomized trials evaluating TyG-GLM6-guided interventions are required to establish causality. Second, CVD ascertainment relied on self-reported physician diagnoses, which may introduce information bias and potential underreporting of events. Lack of adjudication through medical record review or standardized clinical examinations represents a significant limitation. Future studies incorporating objective CVD ascertainment (e.g., medical record linkage, imaging confirmation, biomarker adjudication) would strengthen outcome validity. Third, the study population was predominantly Han Chinese, and generalizability to other ethnic groups and geographic populations remains uncertain. Genetic, environmental, dietary, and healthcare system differences may influence the performance of TyG-GLM6 across populations. Validation in ethnically diverse cohorts from different geographic regions is essential before widespread clinical implementation. Fourth, the modest discriminative performance (AUC: 0.587) reflects the inherent complexity of CVD risk prediction. As discussed above, CVD is a multifactorial endpoint influenced by numerous unmeasured factors. The modest AUC underscores that TyG-GLM6 should serve as a risk stratification tool within high-risk populations rather than a standalone diagnostic marker. Fifth, detailed medication data—particularly use of glucose-lowering agents, statins, and other cardioprotective therapies—were unavailable, potentially introducing confounding by indication. These medications directly influence metabolic parameters and CVD risk, and their absence from adjustment models may bias observed associations. Sixth, residual confounding from unmeasured variables such as genetic predisposition, detailed dietary patterns, physical activity, socioeconomic factors, and psychosocial stress cannot be excluded despite comprehensive covariate adjustment.

The findings of this study have important clinical implications. TyG-GLM6, derived from routine clinical parameters, offers a practical and cost-effective tool for CVD risk stratification in primary care and community health settings serving middle-aged and elderly populations with abnormal glucose metabolism. The linear dose–response relationship facilitates straightforward interpretation and implementation in clinical guidelines. The web-based risk calculator developed in this study provides clinicians with an accessible platform for individualized risk assessment and patient counseling.

## Conclusions

This nationwide prospective cohort study demonstrates that the novel glycolipid composite index TyG-GLM6 exhibits superior and independent predictive value for incident CVD among Chinese middle-aged and elderly individuals with abnormal glucose metabolism. TyG-GLM6 demonstrates a linear dose–response relationship with CVD risk and maintains robust predictive performance across demographic subgroups, outperforming conventional IR surrogate indexes in fully adjusted models. Derived from readily available clinical parameters, TyG-GLM6 represents a practical and cost-effective tool for early risk stratification and personalized cardiovascular prevention strategies in this high-risk population. External validation in an independent clinical cohort corroborates these findings and supports the clinical utility of TyG-GLM6. Future prospective studies in diverse populations are warranted to confirm these findings and evaluate the impact of TyG-GLM6-guided interventions on cardiovascular outcomes.

## Data Availability

The original contributions presented in the study are included in the article/Supplementary Material, further inquiries can be directed to the corresponding authors.
