# Supplementary material for: A novel glycolipid composite index predicting cardiovascular disease in Chinese adults with abnormal glucose metabolism: a nationwide cohort study
Source: Front Cardiovasc Med. 2026 May 14;13:1791890. doi: 10.3389/fcvm.2026.1791890 (PMC13215857; doi:10.3389/fcvm.2026.1791890)
Supplement: Supplementary file 1 [file Datasheet1.docx]

**Supplementary Table 1. Calculation formula of each index**

(1) BMI = weight [kg] / height [m]²

(2) Glycolipid Metabolism 6 Factors (GLM6) = log10[(Age [year] × BMI [kg/m²] × FBG [mg/dL] × TG [mmol/L] × LDL-C [mmol/L]) / HDL-C [mmol/L]];

(3) estimated Glucose Disposal Rate (eGDR) = 21.158 − (0.09 × waist circumference [WC, cm]) − (3.407 × hyperetnsion [yes = 1/no = 0]) − (0.551 × glycosylated hemoglobin A1c [HbA1c, %]);

(4) Triglyceride-glucose (TyG) index = Ln (fasting triglyceride [mg/dL] × fasting glucose [mg/dL]/2);

(5) TyG-WC index = TyG index × WC (cm);

(6) TyG-body mass index (TyG-BMI) index = TyG index × BMI;

(7) TyG-waist-to-height ratio (TyG-WHtR) index = TyG index × WC (cm)/height (cm);

(8) TyG- Glycolipid Metabolism 6 Factors (TyG-GLM6) index = TyG index × GLM6;

(9) Triglyceride-to-high density lipoprotein cholesterol (HDL-C) ratio (TG/HDL-C) = fasting triglyceride (mg/dL)/fasting HDL-C (mg/dL).

(10) Metabolic score for IR (METS-IR) = Ln (2 × fasting glucose [mg/dL] + fasting triglyceride [mg/dL]) × BMI/Ln (fasting HDL-C [mg/dL]).

(11) AIP = log (TG [mg/dL]/ HDL-C [mg/dL])

(12) CVAI (male) = −267.93 + 0.68 × age [y] + 0.03 × BMI + 4.00 × WC [cm] + 22.00 × Ln(TG [mg/dL]) − 16.32 × Ln(HDL-C [mg/dL])

(13) CVAI (female) = −187.32 + 1.71 × age [y] + 0.03 × BMI + 4.00 × WC [cm] + 39.76 × Ln(TG [mg/dL]) − 11.66 × Ln(HDL-C [mg/dL])

**Supplementary Table 2. Univariate and Multivariate Cox Regression Analysis**

| **Index** | **Univariate**  **Cox Regression** | | | **Multivariate**  **Cox Regression** | | |
| --- | --- | --- | --- | --- | --- | --- |
|  | HR | 95%CI | P Value | HR | 95%CI | P Value |
| TyG-GLM6 | 1.026 | 1.017–1.036 | <0.001 | 1.017 | 1.004–1.030 | 0.011 |
| eGDR | 0.905 | 0.878–0.934 | <0.001 | 0.948 | 0.911–0.986 | 0.009 |
| TyG-WC | 1.002 | 1.001–1.002 | <0.001 | 0.998 | 0.997–1.000 | 0.010 |
| CVAI | 1.007 | 1.005–1.008 | <0.001 | 1.007 | 1.004–1.011 | <0.001 |

**Supplementary Table 3. cardiovascular-risk-tool original code**

<!DOCTYPE html>

<html lang="en">

<head>

<meta charset="UTF-8">

<meta name="viewport" content="width=device-width, initial-scale=1.0">

<title>Cardiovascular Risk Prediction Tool for Patients with Glucose Metabolism Disorders</title>

<style>

:root {

--primary: #3498db;

--primary-dark: #2980b9;

--secondary: #2c3e50;

--danger: #e74c3c;

--warning: #f39c12;

--success: #2ecc71;

--light: #ecf0f1;

--gray: #95a5a6;

--dark: #34495e;

}

* {

box-sizing: border-box;

margin: 0;

padding: 0;

}

body {

font-family: 'Segoe UI', Tahoma, Geneva, Verdana, sans-serif;

line-height: 1.6;

color: var(--dark);

background: linear-gradient(135deg, #f5f7fa 0%, #c3cfe2 100%);

min-height: 100vh;

padding: 20px;

}

.container {

max-width: 1000px;

margin: 0 auto;

background: white;

border-radius: 15px;

box-shadow: 0 10px 30px rgba(0, 0, 0, 0.1);

overflow: hidden;

}

header {

background: var(--secondary);

color: white;

padding: 25px;

text-align: center;

position: relative;

}

h1 {

font-size: 28px;

margin-bottom: 10px;

}

.description {

font-size: 16px;

opacity: 0.9;

max-width: 800px;

margin: 0 auto;

}

.scope-notice {

background: rgba(231, 76, 60, 0.1);

border: 1px solid var(--danger);

border-radius: 5px;

padding: 12px 15px;

margin: 15px auto;

max-width: 800px;

font-size: 14px;

color: var(--danger);

}

.form-container {

padding: 25px;

}

.tabs {

display: flex;

margin-bottom: 20px;

border-bottom: 1px solid #ddd;

}

.tab {

padding: 12px 20px;

cursor: pointer;

font-weight: 600;

color: var(--gray);

border-bottom: 3px solid transparent;

transition: all 0.3s;

}

.tab.active {

color: var(--primary);

border-bottom: 3px solid var(--primary);

}

.tab-content {

display: none;

}

.tab-content.active {

display: block;

}

.form-section {

margin-bottom: 25px;

}

.section-title {

font-size: 18px;

color: var(--secondary);

margin-bottom: 15px;

padding-bottom: 8px;

border-bottom: 1px solid #eee;

display: flex;

align-items: center;

}

.section-title svg {

margin-right: 10px;

color: var(--primary);

}

.form-columns {

display: flex;

flex-wrap: wrap;

gap: 20px;

margin-bottom: 15px;

}

.form-group {

flex: 1 1 calc(50% - 20px);

min-width: 250px;

}

label {

display: block;

margin-bottom: 8px;

font-weight: 600;

color: var(--dark);

}

.input-with-unit {

display: flex;

align-items: center;

}

.input-with-unit input {

flex: 1;

}

.unit {

background: var(--light);

padding: 0 12px;

height: 46px;

display: flex;

align-items: center;

justify-content: center;

min-width: 80px;

border: 1px solid #ddd;

border-left: none;

border-radius: 0 4px 4px 0;

}

input, select {

width: 100%;

padding: 12px;

border: 1px solid #ddd;

border-radius: 4px;

font-size: 16px;

transition: border 0.3s;

}

input:focus, select:focus {

border-color: var(--primary);

outline: none;

box-shadow: 0 0 0 3px rgba(52, 152, 219, 0.2);

}

.checkbox-group {

display: flex;

align-items: center;

margin-bottom: 15px;

}

.checkbox-group input {

width: auto;

margin-right: 10px;

}

button {

background: var(--primary);

color: white;

border: none;

padding: 15px 25px;

border-radius: 4px;

cursor: pointer;

font-size: 16px;

font-weight: 600;

width: 100%;

transition: background 0.3s;

display: flex;

align-items: center;

justify-content: center;

}

button svg {

margin-right: 10px;

}

button:hover {

background: var(--primary-dark);

}

.result {

margin-top: 30px;

padding: 25px;

background: var(--light);

border-radius: 10px;

display: none;

}

.result-title {

font-size: 22px;

color: var(--secondary);

margin-bottom: 20px;

text-align: center;

}

.risk-value {

font-size: 42px;

font-weight: bold;

text-align: center;

margin: 20px 0;

color: var(--danger);

}

.risk-bar {

height: 35px;

background-color: #eee;

border-radius: 5px;

margin: 25px 0;

overflow: hidden;

position: relative;

}

.risk-fill {

height: 100%;

background: linear-gradient(to right, var(--success), var(--warning), var(--danger));

width: 0%;

transition: width 1s ease-in-out;

}

.risk-labels {

display: flex;

justify-content: space-between;

margin-top: 10px;

font-size: 14px;

color: var(--dark);

}

.calculated-params {

background: white;

padding: 20px;

border-radius: 8px;

margin-top: 20px;

}

.param-grid {

display: grid;

grid-template-columns: repeat(auto-fit, minmax(200px, 1fr));

gap: 15px;

margin-top: 15px;

}

.param-card {

background: var(--light);

padding: 15px;

border-radius: 8px;

text-align: center;

}

.param-name {

font-size: 14px;

color: var(--gray);

margin-bottom: 5px;

}

.param-value {

font-size: 20px;

font-weight: bold;

color: var(--secondary);

}

.points-info {

margin-top: 25px;

font-size: 14px;

color: var(--dark);

background: white;

padding: 15px;

border-radius: 8px;

}

@media (max-width: 768px) {

.form-group {

flex: 1 1 100%;

}

.param-grid {

grid-template-columns: 1fr;

}

}

.required::after {

content: "*";

color: var(--danger);

margin-left: 4px;

}

.info-icon {

display: inline-block;

width: 18px;

height: 18px;

border-radius: 50%;

background: var(--primary);

color: white;

text-align: center;

line-height: 18px;

font-size: 12px;

margin-left: 5px;

cursor: help;

position: relative;

}

.info-icon:hover::after {

content: "This tool is for cardiovascular risk prediction in patients with glucose metabolism disorders";

position: absolute;

bottom: 100%;

left: 50%;

transform: translateX(-50%);

width: 250px;

padding: 10px;

background: var(--dark);

color: white;

border-radius: 5px;

font-size: 12px;

margin-bottom: 5px;

z-index: 10;

}

</style>

</head>

<body>

<div class="container">

<header>

<h1>Cardiovascular Risk Prediction Tool</h1>

<p class="description">Assess cardiovascular disease risk probability based on your physiological indicators and test results</p>

<div class="scope-notice">

Scope: This tool is applicable for cardiovascular disease risk prediction in patients with glucose metabolism disorders (including prediabetes and diabetes).

Assessment results are for reference only and cannot replace professional medical diagnosis.

</div>

</header>

<div class="form-container">

<form id="riskForm">

<div class="tabs">

<div class="tab active" data-tab="basic">Basic Information</div>

<div class="tab" data-tab="clinical">Clinical Indicators</div>

<div class="tab" data-tab="blood">Blood Tests</div>

</div>

<div class="tab-content active" id="basic-tab">

<div class="form-columns">

<div class="form-group">

<label for="age" class="required">Age</label>

<div class="input-with-unit">

<input type="number" id="age" min="18" max="100" required>

<div class="unit">years</div>

</div>

</div>

<div class="form-group">

<label for="gender" class="required">Gender</label>

<select id="gender" required>

<option value="">Please select</option>

<option value="male">Male</option>

<option value="female">Female</option>

</select>

</div>

</div>

<div class="form-columns">

<div class="form-group">

<label for="height" class="required">Height</label>

<div class="input-with-unit">

<input type="number" id="height" min="100" max="250" step="0.1" required>

<div class="unit">cm</div>

</div>

</div>

<div class="form-group">

<label for="weight" class="required">Weight</label>

<div class="input-with-unit">

<input type="number" id="weight" min="30" max="200" step="0.1" required>

<div class="unit">kg</div>

</div>

</div>

</div>

<div class="form-columns">

<div class="form-group">

<label for="wc" class="required">Waist Circumference</label>

<div class="input-with-unit">

<input type="number" id="wc" min="50" max="150" step="0.1" required>

<div class="unit">cm</div>

</div>

</div>

<div class="form-group">

<label for="hypertension" class="required">Hypertension</label>

<select id="hypertension" required>

<option value="">Please select</option>

<option value="1">Yes</option>

<option value="0">No</option>

</select>

<span class="info-icon">i</span>

</div>

</div>

</div>

<div class="tab-content" id="clinical-tab">

<div class="form-columns">

<div class="form-group">

<label for="hba1c" class="required">HbA1c</label>

<div class="input-with-unit">

<input type="number" id="hba1c" min="4" max="15" step="0.1" required>

<div class="unit">%</div>

</div>

</div>

<div class="form-group">

<label for="fbg" class="required">Fasting Blood Glucose</label>

<div class="input-with-unit">

<input type="number" id="fbg" min="3.9" max="20" step="0.1" required>

<div class="unit">mmol/L</div>

</div>

</div>

</div>

</div>

<div class="tab-content" id="blood-tab">

<div class="form-columns">

<div class="form-group">

<label for="tg" class="required">Triglycerides</label>

<div class="input-with-unit">

<input type="number" id="tg" min="0.3" max="10" step="0.01" required>

<div class="unit">mmol/L</div>

</div>

</div>

<div class="form-group">

<label for="hdl" class="required">HDL Cholesterol</label>

<div class="input-with-unit">

<input type="number" id="hdl" min="0.5" max="3" step="0.01" required>

<div class="unit">mmol/L</div>

</div>

</div>

</div>

<div class="form-columns">

<div class="form-group">

<label for="ldl" class="required">LDL Cholesterol</label>

<div class="input-with-unit">

<input type="number" id="ldl" min="1" max="6" step="0.01" required>

<div class="unit">mmol/L</div>

</div>

</div>

</div>

</div>

<button type="submit">

<svg xmlns="http://www.w3.org/2000/svg" width="20" height="20" viewBox="0 0 24 24" fill="none" stroke="currentColor" stroke-width="2" stroke-linecap="round" stroke-linejoin="round">

<path d="M21 15v4a2 2 0 0 1-2 2H5a2 2 0 0 1-2-2v-4"></path>

<polyline points="7 10 12 15 17 10"></polyline>

<line x1="12" y1="15" x2="12" y2="3"></line>

</svg>

Calculate Cardiovascular Risk Probability

</button>

</form>

<div id="result" class="result">

<h2 class="result-title">Cardiovascular Risk Assessment Result</h2>

<p>Based on your inputs, your cardiovascular disease risk probability is:</p>

<div class="risk-value" id="riskValue">0.0</div>

<div class="risk-bar">

<div class="risk-fill" id="riskFill"></div>

</div>

<div class="risk-labels">

<span>Low Risk (0.1)</span>

<span>Medium Risk (0.3)</span>

<span>High Risk (0.5)</span>

<span>Very High Risk (0.7)</span>

</div>

<div class="calculated-params">

<h3>Calculated Parameters</h3>

<div class="param-grid">

<div class="param-card">

<div class="param-name">TyG_GLM6</div>

<div class="param-value" id="paramTyG_GLM6">0.00</div>

</div>

<div class="param-card">

<div class="param-name">TyG_WC</div>

<div class="param-value" id="paramTyG_WC">0.00</div>

</div>

<div class="param-card">

<div class="param-name">eGDR</div>

<div class="param-value" id="paramEGDR">0.00</div>

</div>

<div class="param-card">

<div class="param-name">CVAI</div>

<div class="param-value" id="paramCVAI">0.00</div>

</div>

</div>

</div>

<div class="points-info">

<p>Points for each indicator: <span id="pointsBreakdown"></span></p>

<p>Total points: <span id="totalPoints"></span></p>

</div>

</div>

</div>

</div>

<script>

// Tab switching functionality

document.querySelectorAll('.tab').forEach(tab => {

tab.addEventListener('click', () => {

// Remove all active tabs and content

document.querySelectorAll('.tab').forEach(t => t.classList.remove('active'));

document.querySelectorAll('.tab-content').forEach(c => c.classList.remove('active'));

// Activate current tab and content

tab.classList.add('active');

const tabId = tab.getAttribute('data-tab') + '-tab';

document.getElementById(tabId).classList.add('active');

});

});

// Form submission handling

document.getElementById('riskForm').addEventListener('submit', function(e) {

e.preventDefault();

// Get input values

const age = parseFloat(document.getElementById('age').value);

const gender = document.getElementById('gender').value;

const height = parseFloat(document.getElementById('height').value);

const weight = parseFloat(document.getElementById('weight').value);

const wc = parseFloat(document.getElementById('wc').value);

const hypertension = parseInt(document.getElementById('hypertension').value);

const hba1c = parseFloat(document.getElementById('hba1c').value);

const fbg = parseFloat(document.getElementById('fbg').value); // Unit is mmol/L

const tg = parseFloat(document.getElementById('tg').value); // Unit is mmol/L

const hdl = parseFloat(document.getElementById('hdl').value);

const ldl = parseFloat(document.getElementById('ldl').value);

// Calculate BMI

const heightM = height / 100;

const bmi = weight / (heightM * heightM);

// Calculate TyG (using mmol/L units)

// Note: TyG formula requires mg/dL units, so conversion is needed

const tgMgDl = tg * 88.57; // Convert TG to mg/dL

const fbgMgDl = fbg * 18; // Convert FBG to mg/dL

const tyg = Math.log((tgMgDl * fbgMgDl) / 2);

// Calculate four parameters

const tygWc = tyg * wc;

const egdr = 21.158 - (0.09 * wc) - (3.407 * hypertension) - (0.551 * hba1c);

let cvai;

// TG unit is already mmol/L, no conversion needed

if (gender === 'male') {

cvai = -267.93 + (0.68 * age) + (0.03 * bmi) + (4.00 * wc) +

(22.00 * Math.log10(tg)) - (16.32 * hdl);

} else {

cvai = -187.32 + (1.71 * age) + (4.23 * bmi) + (1.12 * wc) +

(39.76 * Math.log10(tg)) - (11.66 * hdl);

}

// Calculate TyG_GLM6 (using mmol/L units)

// Note: FBG needs to be converted to mg/dL

const tygGlm6 = tyg * Math.log10((age * bmi * fbgMgDl * tg * ldl) / hdl);

// Display calculated parameters

document.getElementById('paramTyG_GLM6').textContent = tygGlm6.toFixed(2);

document.getElementById('paramTyG_WC').textContent = tygWc.toFixed(2);

document.getElementById('paramEGDR').textContent = egdr.toFixed(2);

document.getElementById('paramCVAI').textContent = cvai.toFixed(2);

// Calculate points for each parameter

const pointsTyG_GLM6 = calculateTyG_GLM6Points(tygGlm6);

const pointsTyG_WC = calculateTyG_WCPoints(tygWc);

const pointsEGDR = calculateEGDRPoints(egdr);

const pointsCVAI = calculateCVAIPoints(cvai);

// Calculate total points

const totalPoints = pointsTyG_GLM6 + pointsTyG_WC + pointsEGDR + pointsCVAI;

// Calculate risk probability based on total points

const risk = calculateRisk(totalPoints);

// Display results

document.getElementById('riskValue').textContent = risk.toFixed(3);

document.getElementById('pointsBreakdown').textContent =

`TyG_GLM6: ${pointsTyG_GLM6.toFixed(1)}, TyG_WC: ${pointsTyG_WC.toFixed(1)}, eGDR: ${pointsEGDR.toFixed(1)}, CVAI: ${pointsCVAI.toFixed(1)}`;

document.getElementById('totalPoints').textContent = totalPoints.toFixed(1);

// Update risk bar

const riskFill = document.getElementById('riskFill');

const percentage = (risk - 0.1) / (0.7 - 0.1) * 100;

riskFill.style.width = Math.min(Math.max(percentage, 0), 100) + '%';

// Show result section

document.getElementById('result').style.display = 'block';

// Scroll to result section

document.getElementById('result').scrollIntoView({ behavior: 'smooth' });

});

// Convert parameters to points based on nomogram

function calculateTyG_GLM6Points(value) {

// Based on nomogram: 30→0 points, 45→25 points, 60→50 points, 75→100 points

if (value <= 30) return 0;

if (value >= 75) return 100;

if (value <= 45) {

return (value - 30) / (45 - 30) * 25;

} else if (value <= 60) {

return 25 + (value - 45) / (60 - 45) * 25;

} else {

return 50 + (value - 60) / (75 - 60) * 50;

}

}

function calculateTyG_WCPoints(value) {

// Based on nomogram: 1300→0 points, 1100→20 points, 900→40 points, 700→60 points, 500→80 points, 300→90 points, 100→100 points

if (value >= 1300) return 0;

if (value <= 100) return 100;

if (value >= 1100) {

return (1300 - value) / (1300 - 1100) * 20;

} else if (value >= 900) {

return 20 + (1100 - value) / (1100 - 900) * 20;

} else if (value >= 700) {

return 40 + (900 - value) / (900 - 700) * 20;

} else if (value >= 500) {

return 60 + (700 - value) / (700 - 500) * 20;

} else if (value >= 300) {

return 80 + (500 - value) / (500 - 300) * 10;

} else {

return 90 + (300 - value) / (300 - 100) * 10;

}

}

function calculateEGDRPoints(value) {

// Based on nomogram: 18→0 points, 14→25 points, 10→50 points, 6→75 points, 2→100 points

if (value >= 18) return 0;

if (value <= 2) return 100;

if (value >= 14) {

return (18 - value) / (18 - 14) * 25;

} else if (value >= 10) {

return 25 + (14 - value) / (14 - 10) * 25;

} else if (value >= 6) {

return 50 + (10 - value) / (10 - 6) * 25;

} else {

return 75 + (6 - value) / (6 - 2) * 25;

}

}

function calculateCVAIPoints(value) {

// Based on nomogram: -200→0 points, -150→10 points, -100→20 points, -50→30 points, 0→40 points,

// 50→50 points, 100→60 points, 150→70 points, 200→80 points, 250→90 points, 300→95 points, 350→100 points

if (value <= -200) return 0;

if (value >= 350) return 100;

if (value <= -150) {

return (value - (-200)) / (-150 - (-200)) * 10;

} else if (value <= -100) {

return 10 + (value - (-150)) / (-100 - (-150)) * 10;

} else if (value <= -50) {

return 20 + (value - (-100)) / (-50 - (-100)) * 10;

} else if (value <= 0) {

return 30 + (value - (-50)) / (0 - (-50)) * 10;

} else if (value <= 50) {

return 40 + (value - 0) / (50 - 0) * 10;

} else if (value <= 100) {

return 50 + (value - 50) / (100 - 50) * 10;

} else if (value <= 150) {

return 60 + (value - 100) / (150 - 100) * 10;

} else if (value <= 200) {

return 70 + (value - 150) / (200 - 150) * 10;

} else if (value <= 250) {

return 80 + (value - 200) / (250 - 200) * 10;

} else if (value <= 300) {

return 90 + (value - 250) / (300 - 250) * 5;

} else {

return 95 + (value - 300) / (350 - 300) * 5;

}

}

// Calculate risk probability based on total points

function calculateRisk(totalPoints) {

// Based on nomogram: total points from 0 to 160 correspond to risk probability from 0.1 to 0.7

const minPoints = 0;

const maxPoints = 160;

const minRisk = 0.1;

const maxRisk = 0.7;

// Ensure total points are within valid range

const clampedPoints = Math.max(minPoints, Math.min(maxPoints, totalPoints));

// Linear interpolation to calculate risk

return minRisk + (maxRisk - minRisk) * (clampedPoints / maxPoints);

}

</script>

</body>

</html>

**User Interface**


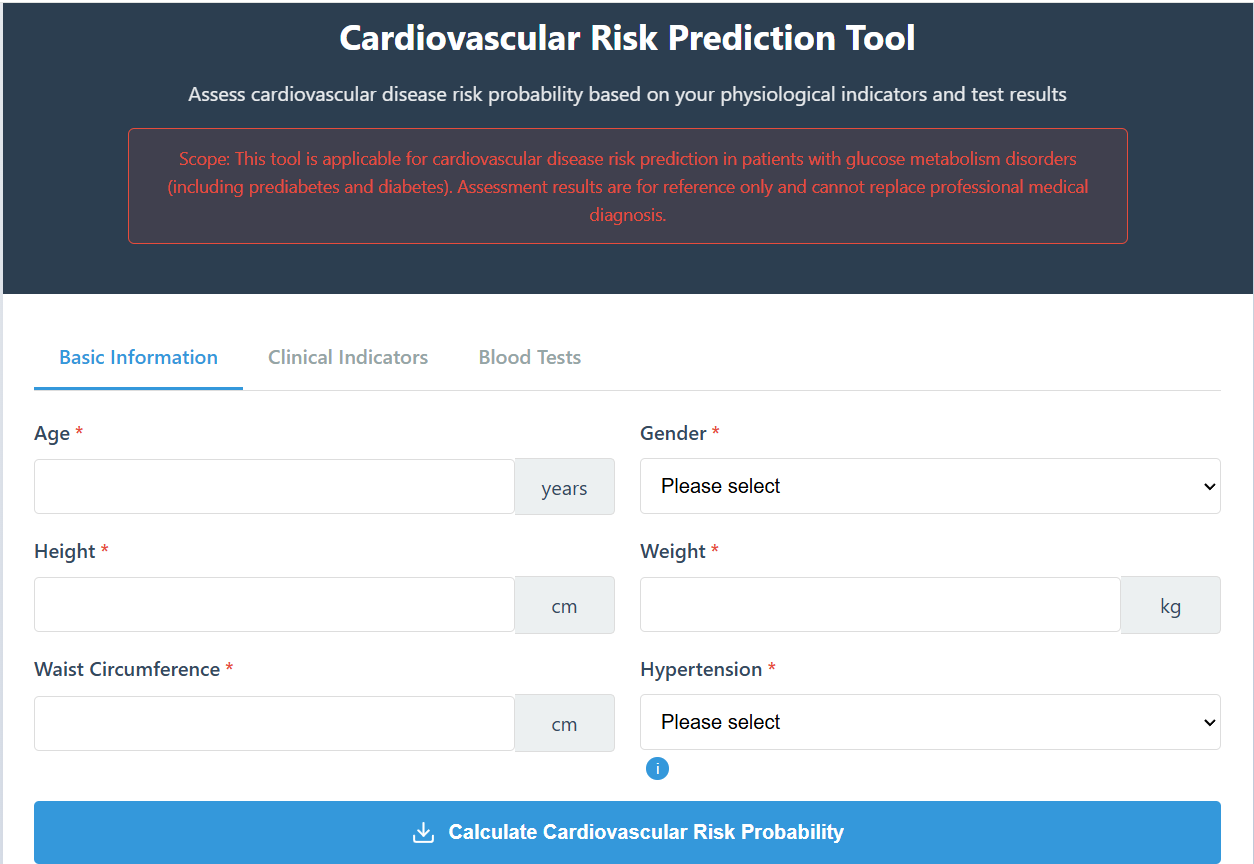


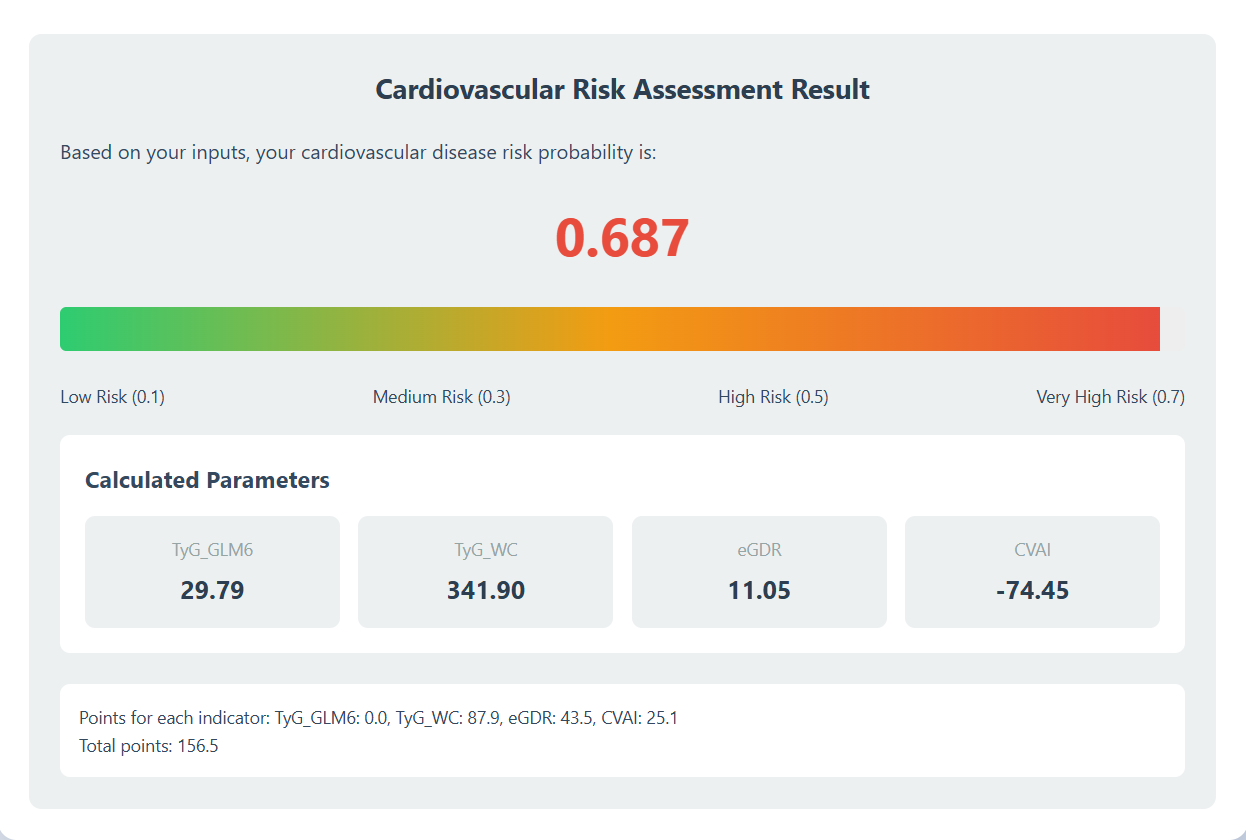


**Supplementary Table 4 Clinical Data Population Characteristics**

| Variables | Total  (n = 2105) | NO CVD  (n = 1046) | CVD  (n = 1059) | *P* |
| --- | --- | --- | --- | --- |
|  |  |  |  |  |
| Age | 65.46 ± 12.71 | 61.49 ± 12.54 | 69.37 ± 11.62 | **<.001** |
| HbA1c | 7.93 ± 1.54 | 7.55 ± 1.22 | 8.31 ± 1.71 | **<.001** |
| GLU | 7.70 ± 2.91 | 7.32 ± 1.96 | 8.07 ± 3.58 | **<.001** |
| TG | 1.61 ± 0.85 | 1.61 ± 0.78 | 1.62 ± 0.91 | 0.689 |
| LDL | 2.55 ± 0.89 | 2.80 ± 0.83 | 2.31 ± 0.88 | **<.001** |
| HDL | 1.08 ± 0.31 | 1.18 ± 0.30 | 0.99 ± 0.29 | **<.001** |
| TyG-GLM6 | 40.36 ± 5.73 | 39.90 ± 5.29 | 40.81 ± 6.10 | **<.001** |
| Gender, n(%) |  |  |  | 0.126 |
| Female | 799 (37.96) | 380 (36.33) | 419 (39.57) |  |
| Male | 1306 (62.04) | 666 (63.67) | 640 (60.43) |  |
